# Sulfakinins influence lipid composition and insulin-like peptides level in oenocytes of *Zophobas atratus* beetles

**DOI:** 10.1007/s00360-021-01398-2

**Published:** 2021-08-20

**Authors:** M. Szymczak-Cendlak, M. Gołębiowski, S. Chowański, J. Pacholska-Bogalska, P. Marciniak, G. Rosiński, M. Słocińska

**Affiliations:** 1 Department of Animal Physiology and Developmental Biology, Faculty of Biology, Adam Mickiewicz University in Poznań, ul. Uniwersytetu Poznańskiego 6, 61-614 Poznań, Poland; 2 Laboratory of Analysis of Natural Compounds, Department of Environmental Analysis, Faculty of Chemistry, University of Gdańsk, ul. Wita Stwosza 63, 80-308 Gdańsk, Poland

**Keywords:** Oenocytes, Sulfated and nonsulfated sulfakinin, Sulfakinin receptor, Insulin-like peptides, *Zophobas atratus*, Beetles, Insects

## Abstract

Insect sulfakinins are pleiotropic neuropeptides with the homology to vertebrate gastrin/cholecystokinin peptide family. They have been identified in many insect species and affect different metabolic processes. They have a strong influence on feeding and digestion as well as on carbohydrate and lipid processing. Our study reveals that sulfakinins influence fatty acids composition in *Zophobas atratus* oenocytes and regulate insulin-like peptides (ILPs) level in these cells. Oenocytes are cells responsible for maintenance of the body homeostasis and have an important role in the regulation of intermediary metabolism, especially of lipids. To analyze the lipid composition in oenocytes after sulfakinins injections we used gas chromatography combined with mass spectrometry and for ILPs level determination an immunoenzymatic test was used. Because sulfakinin peptides and their receptors are the main components of sulfakinin signaling, we also analyzed the presence of sulfakinin receptor transcript (SKR2) in insect tissues. We have identified for the first time the sulfakinin receptor transcript (SKR2) in insect oenocytes and found its distribution more widespread in the peripheral tissues (gut, fat body and haemolymph) as well as in the nervous and neuro-endocrine systems (brain, ventral nerve cord, *corpora cardiaca/corpora allata* CC/CA) of *Z. atratus* larvae. The presence of sulfakinin receptor transcript (SKR2) in oenocytes suggests that observed effects on oenocytes lipid and ILPs content may result from direction action of these peptides on oenocytes.

**Supplementary Information:**

The online version contains supplementary material available at 10.1007/s00360-021-01398-2.

## Introduction

Sulfakinins (SKs) are insect neuropeptides that display sequence homology with the vertebrate gastrin/cholecystokinin peptide family (Nachman et al. 1986b). Members of the SKs family have been identified in various insects, including cockroaches (Nachman et al. 1986a, b; Veenstra 1989), locusts (Schoofs et al. 1990), flies (Duve et al. 1994; Fonagy et al. 1992), crickets (Meyering-Vos and Muller 2007) and beetles (Maestro et al. 2001; Marciniak et al. 2010; Weaver and Audsley 2008). Sulfakinins, according to their name, contain a sulfated or nonsulfated tyrosyl residue in their conserved C-terminal heptapeptide. Both forms, sulfated (sSK) and nonsulfated (nsSK), are biologically active and affect different processes.

The first peptide hormone of this family was isolated from the cockroach, *Leucophea maderae* (Nachman et al. 1986b). This peptide increased frequency of spontaneous contractions of cockroach hindgut, and sulfated tyrosine was reported to be required for this activity. Sulfated sulfakinins affect also other physiological functions: foregut (Maestro et al. 2001) and heart contractions (Nichols et al. 2009), food intake (Meyering-Vos and Muller 2007; Nachman et al. 2005; Wei et al. 2000; Yu et al. 2013), digestion (Harshini et al. 2002; Nachman et al. 1997; Zels et al. 2015) and carbohydrate ingestion (Downer et al. 2007). Nonsulfated sulfakinin influences the contractility of ejaculatory duct, oviduct, (Marciniak et al. 2011), heart (Marciniak et al. 2011; Nichols et al. 2009), foregut (Nichols 2007) and are engaged in the regulation of carbohydrate and lipid metabolism in fat body and haemolymph (Marciniak et al. 2011; Słocińska et al. 2015a, b) as well as energy transformations within the insect body (Słocińska et al. 2016). Both sulfakinins, sSK and nsSK, modulate the fatty acid level and composition in fat body and haemolymph (Słocińska et al. 2019). Moreover, recently, Słocińska et al. (2020) demonstrated that either sSKs or nsSK influence cellular homeostasis by the regulation of insulin-like peptides (ILPs) and carbohydrates level in insects.

Essential components of the sulfakinin activation in diverse biological processes occurring in insects are sulfakinin receptors (SKRs) belonging to G-protein-coupled receptors family (GPCRs). First two insect SKRs, named DSKR1 and DSKR2, were predicted and cloned in *Drosophila melanogaster* based on their sequence similarity to the cholecystokinin (CCK) receptors (Hauser et al. 2006; Kubiak et al. 2002). Next, the perisulfakinin receptor (PSKR) was identified within the central nervous system in *Periplaneta americana* (Wicher et al. 2007). Based on the genome sequence in *Tribolium castaneum* two SKR genes were predicted and characterized (Hauser et al. 2008). Another research with *T. castaneum* were performed to analyze the signaling properties and pharmacology of its two sulfakinin receptors (Yu et al. 2013; Zels et al. 2014) which were functional characterized (SKR2) by Yu and Smagghe (2014). Moreover, in 2019, the cDNA sequences encoding two SKRs in *Rhodnius prolixus* were identified and their intracellular signaling pathways were analyzed (Bloom et al. 2019). Obtained results indicated that both sulfated Rhopr-SKs activate two Rhopr-SKRs. They activate the intracellular Ca^2+^ second messenger pathway, but not the cyclic AMP pathway (Bloom et al. 2019). Recently, the SKRs were identified and characterized in *Tenebrio molitor* beetle (Słocińska et al. 2020). SKR transcript distribution in the insect's body is almost ubiquitous. In relation to the tissue-dependency, the most abundant level of SKR transcript was detected in the nervous system and much lower level in the peripheral tissues and organs as fat body, gut, haemolymph, salivary glands, testes, ovaries, heart and Malpighian tubules (Bloom et al. 2019; Słocińska et al. 2020; Yu et al. 2013; Zels et al. 2014).

Although the pleiotropic functions of sulfakinins were confirmed for different tissues and organs, their action in oenocytes is still unknown. Oenocytes are cells of ectodermal origin scattered among epidermal cells, also forming clusters near spiracles, close to large trachea and among the cells of the adipose tissue (Fan et al. 2003; Szymczak and Rosiński 2019). For example, oenocytes of *R. prolixus* nymphs are linked to the epidermis by prolonged transport of synthesized lipids to epidermal cells (Wigglesworth 1988), and in *Calpodes ethlius* larvae oenocytes are located close to epidermal wax glands, suggesting that these cells participate in the synthesis of wax precursors (Locke 1969). Despite studies showing oenocytes as lipid-processing cells (Gutierrez et al. 2007; Martins et al. 2011) their physiological role is not limited to this function (Martins and Ramalho-Ortigão 2012). These cells have been shown to participate in organismal homeostasis (Balabanidou et al. 2016; Clark and Dahm 1973; Lycett et al. 2006; Marriel et al. 2016), in the synthesis of some long hydrocarbon chains of sex pheromones (Grigoraki et al. 2020; Wicker-Thomas et al. 2009) and other cuticular components (Fan et al. 2003), and in the initiation of immune response (Martins et al. 2011). Oenocytes also play a role in the differentiation of neurons during embryogenesis of *D. melanogaster* flies through the secretion of semaforin (Sema2a) a peptide that causes axon elongation (Bates and Whitington 2007). Other studies have shown that oenocytes produce lipids that are involved in the protection of the insect body from water losses (Lockey 1988). Oenocytes express many genes encoding proteins involved in lipid metabolism similarly to mammalian hepatocytes (Gutierrez et al. 2007). Moreover, they are responsible for low lipid content in haemolymph and its mobilization from the fat body depending on the energetic requirements of other tissues (Gutierrez et al. 2007).

Considering the unknown role of sulfakinins in insect oenocytes and engagement of sulfakinin signaling in lipid and carbohydrate metabolism, we aimed to analyze the effect of sSK and nsSK on fatty acids profiles and ILPs level in oenocytes of feeding larvae of *Zophobas atratus* beetle. For analysis of changes in fatty acid and other lipid components composition we applied gas chromatography combined with mass spectrometry, and immunoenzymatic test for determination of changes in the ILPs level. We also performed reverse transcription PCR (RT-PCR) to analyze distribution profile of sulfakinin receptor (SKR2) of *Z. atratus* beetle in different tissues and organs of larvae. Through these experiments, we aimed to examine the involvement of the signaling pathways focusing on sulfakinins and their receptors in regulation of physiological state of oenocytes. Moreover, the combination of above analysis allows us to know, if observed changes might be a direct effect of sulfakinin action on oenocytes.

## Materials and methods

### Peptides

Sulfakinins: the sulfated (pETSDDY(SO3H)GHLRFa) and nonsulfated (pETSDDYGHLRFa) were synthesized according to the Fmoc procedure described previously by Marciniak et al. (2011) and Słocińska et al. (2019).

### Insects and peptide application

*Z. atratus* larvae were reared according to the Quennedy procedure (Quennedey et al. 1995). Insects were derived from a culture maintained at the Department of Animal Physiology and Development, AMU in Poznań. Larvae were grown in containers filled with layers of peat mixed with gardening soil and sawdust and were fed with fresh lettuce leaves and carrot slices. Food was occasionally enriched with a small amount of curd and powdered milk as well as the addition of brewer’s yeast and daphnia to reduce the phenomenon of cannibalism. Containers with larvae were kept in a breeding room at constant temperature conditions of 27–28 °C, relative humidity 60 ± 5% and photoperiod 8:16 h of light to dark.

All experiments were carried out on oenocytes isolated from at least 20 larvae (lipid analysis) or ten larvae (ILPs analysis) for each sample. The procedure of cell collection was as follows: the first and the last segments of larval body were cut off and the midline was cut open. The fat body, digestive organs and Malpighian tubules were removed from the body. The oenocytes were mechanically separated from the body using fine scissors and collected in Eppendorf tubes. The gently separation of cells was conducted under the stereoscopic microscope. For experiments larvae weighting 600–800 mg were used. Control insects were treated with 4 µl of Ringer’s solution (RS) (274 mM NaCl, 19 mM KCl, 9 mM CaCl_2_) by injection and hormone-treated insects were injected with 20 pmol of sSK or nsSK in 4 µl of RS. Injections were performed through the intersegmental membrane between the second and the third abdominal segments in the directions of head. The peptide dose and the time point of sample collections were determined according to the previously prepared experiments. Oenocytes were collected 24 h after the hormones or RS injections for lipid analysis and 2 h after injection for ILPs level determination. All larvae were anesthetized with CO_2_ before injection or cell collection.

### RNA extraction and SKR2 transcript profiles

Dissected tissues (brain, ventral nerve cord, fat body, gut, haemolymph, oenocytes, *corpora cardiaca/corpora allata*) and whole body of larvae were homogenized in RNA lysis buffer (Zymo Research, USA) using a pellet homogenizer. The samples of tissues were prepared from at least ten individuals, whereas sample of whole body from three individuals. Then, the tissues were immediately frozen in liquid nitrogen and stored at − 80 °C. Total RNA was extracted following the manufacturer’s instructions using a Quick-RNA® Mini Prep kit (Zymo Research, USA). To determine the RNA concentration, we used Synergy H1 Hybrid Multi-Mode Microplate Reader (BioTek, USA). The same amount of isolated RNA was used for cDNA synthesis with the RivertAid™ Reverse Transcriptase kit (Thermo-Fisher, USA). A T100^TM^ Marone et al. (2001) Two biological and four technical repetitions were done for each research variant.

The primer pair for Zopat-SKR2 were designed using Primer3 software (Untergasser et al. 2012) with the following sequence: Forward: 5′–CGCCTACATCACCCTGCAT–3′ and Reverse: 5′–AGTCCTGGGACCAGTTGTGA–3′. The primers were synthetized by the Institute of Biochemistry and Biophysics of the Polish Academy of Science (Warsaw, Poland). PCR was performed in a 10 μL reaction volume with Thermo-Fisher Scientific (USA) reagents: DNase/RNase-free water, DreamTaq™ Green Buffer, dNTP, Dream Taq™ DNA polymerase and then primers and cDNA was added. After PCR, the products were analyzed by electrophoresis using 2.5% TAE agarose gel stained with ethidium bromide and the bands were visualized with ChemiDoc™ Touch (Bio-Rad, USA). “No template control” and “no RT control” reactions were included in the analysis to ensure that there was no foreign DNA or genomic DNA contamination. In each analysis, RpS18 gene was used as an additional control.

### Sequence analysis of Zopat-SKR2

To obtain Zopat-SKR2 sequence, *Z. atratus* brain and retrocerebral complex transcriptome (SRR11178058 and SRR11178059, BioProject PRJNA608269) was searched by tblastn algorithm with *T. castaneum* SKR sequence receptor (XP_972750.1). The established protein sequence of the Zopat-SKR was analyzed for the presence of putative transmembrane regions with the software programs PSIPRED–MEMSAT (https://bioinf.cs.ucl.ac.uk/psipred/) (Nugent and Jones 2009). Protein sequence alignment of Zopat-SKRs with other Tenebrionid SKRs was performed with Clustal W (https://embnet.vital-it.ch/software/ClustalW.html). All alignments and similarity analysis were visualized with the usage of Jalview and Ugene software.

### Extraction of lipids and gas chromatography–mass spectrometry (GC–MS)

For each sample, oenocytes (ca 30 mg) from 20 insects were isolated, transferred to 100 µl of distilled water and then evaporated at 30 °C under vacuum, and the dry mass of samples was weighted. In the next step, dried samples were dissolved and extracted in 1.5 ml of dichloromethane and were shaken for 30 min. Next, the procedure of preparation was the same as described previously by Słocińska et al. (2020). Briefly, solvent was removed from samples under a gentle stream of nitrogen, and components of extracts were silylated with 100 μl of a mixture of 99% bis(trimethylsilyl)acetamide and 1% chlorotrimethylsilane at 100 °C for 1 h on the day of analysis. Gas chromatography–mass spectrometry (GC–MS) measurements were performed as described previously by Słocińska et al. (2019).

### Immunoenzymatic determination of the insulin-like peptides level in oenocytes

The insulin-like peptides level in oenocytes extracts was determined with ELISA Insulin Kit (DRG Instruments GmbH, Germany. Cat. # EIA2935) as described previously by Chowański et al. (2018) and Słocińska et al. (2020) with some modification. Briefly, for each sample the oenocytes from ten insects were isolated 2 h after hormones or RS injection and collected to 30 μL of ice-cold saline and next, the samples were shaken for 15 min (1400 rpm at 4 °C) on a thermomixer (Eppendorf, Germany) and stored at − 20 °C till measurement. Three biological and one technical repetitions were done for each research variant. Before analysis, the samples were centrifuged at 10,000 ×*g* at 4 °C for 10 min and the concentration of soluble proteins was measured using infrared spectrometer Direct Detect (Merck). Next, 25 μL of samples were added to the wells of ELISA Insulin Kit and incubated with Enzyme Conjugate for 30 min. Then, after removal of the solution, the wells were washed three times with a wash buffer, and the Enzyme Complex was added. After 30 min of incubation and triple washing, the substrate solution was added, and the samples were incubated for the next 15 min. Finally, the stop reaction solution was added, and the absorbance was measured (*λ* = 450 nm) with Tecan Sunrise™ absorbance microplate reader. The concentration of the ILPs was calculated based on a standard curve and expressed as ng of ILPs per mg of soluble proteins in a sample.

### Statistical analysis

All data are presented as the mean values ± SD of the indicated number of individuals (*n*). The statistical significance of differences between the mean values of the control and experimental groups of insects was determined using one-way ANOVA analysis. The statistical analysis was performed using Graph Pad Prism software. Differences were considered statistically significant if *p* < 0.05 (*), *p* < 0.01 (**) and *p* < 0.00 (***).

## Results

### Analysis of Zopat-SKR2 sequence

**Fig. 1:**
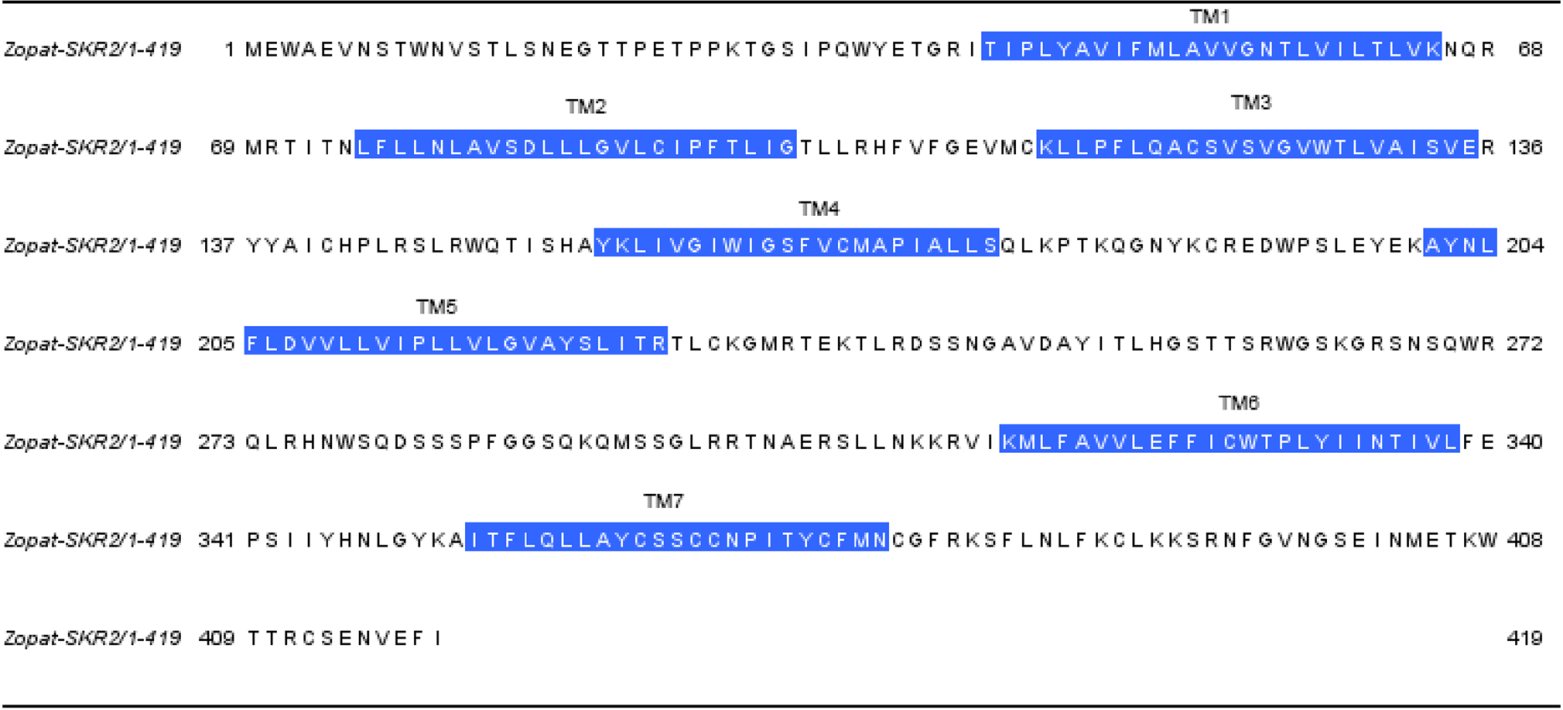
Amino acid sequences of SKR2 deduced from transcriptomic data of brain and retrocerebral complex of adult *Z. atratus* beetle. Predicted transmembrane regions (TM) are highlighted in blue (colour figure online)

**Fig. 2:**
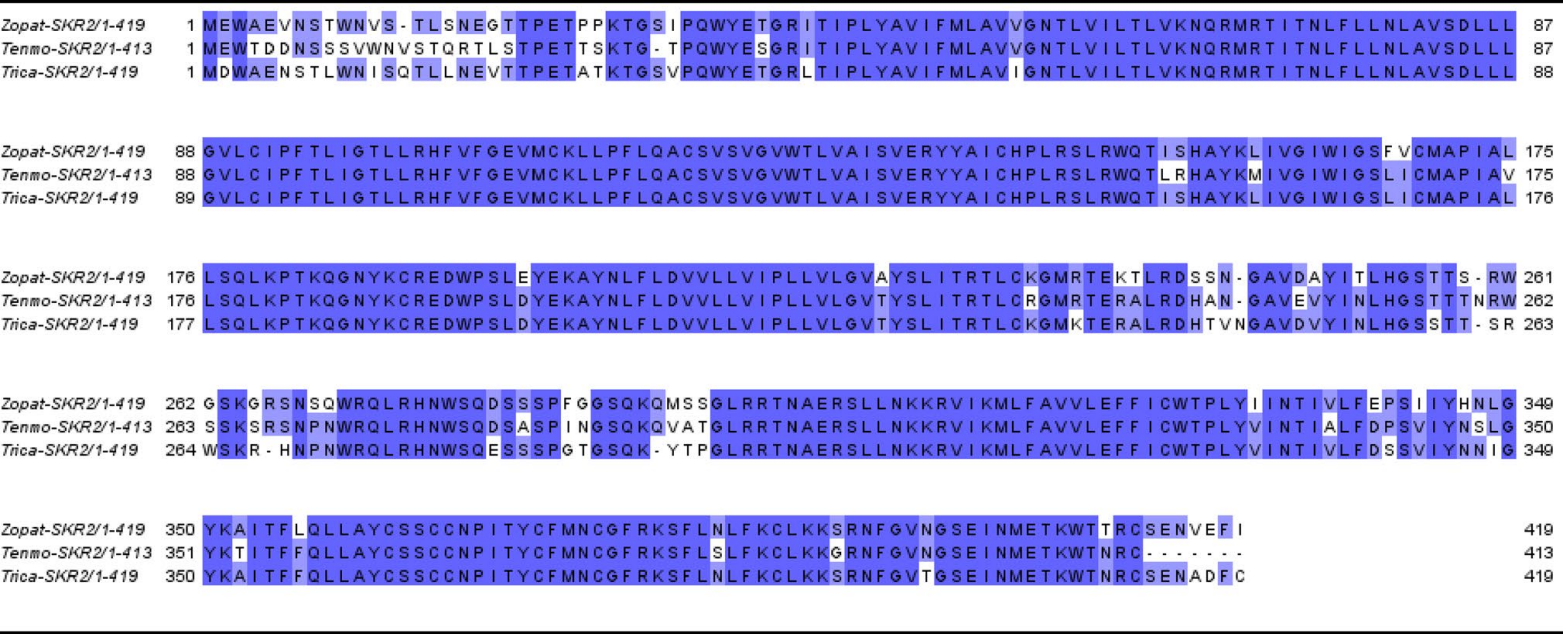
Sequence alignment of SKR2 amino acid sequences in Tenebrionid beetles. Identical and conserved amino acids across sequences are color coded in dark and light blue, respectively (colour figure online)

Based on the BLAST search with local database, transcriptomic assembly of *Z. atratus* brain and retrocerebral complex yielded an open reading frame—1257 bp which encodes a putative sulfakinin receptor 2 (Zopat-SKR-2). It displays the seven transmembrane domains typical for GPCRs (Bass et al. 2014) with N-terminal ligand binding tail and C-terminal intracellular region (Fig. 1, Suppl. Fig. 1). Protein sequence alignment with other closely related Tenebrionid beetles shows a very high degree of identity and similarity (Fig. 2). As expected, slightly higher variability was observed in N-terminal and C-terminal regions (Fig. 2). Practically 100% identity was observed in sequences of transmembrane helices (Fig. 2). The predicted post-translational modifications of the receptor protein include the typical glycosylation of N-terminal region and extracellular loops, phosphorylation by protein kinase C and cAMP and cGMP dependent kinases and palmitoylation at the C-terminal tail (Suppl. Fig. 1).

### Transcript distribution profile of Zopat-SKR in different tissues of larvae

**Fig. 3:**
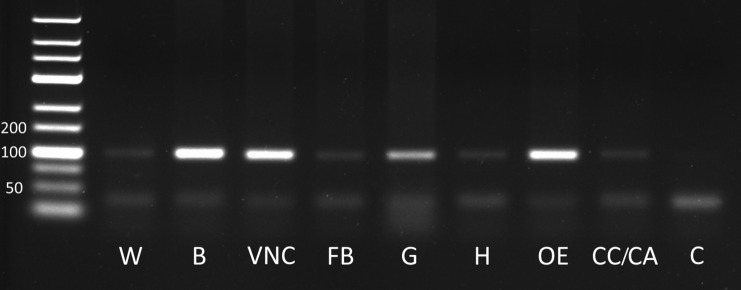
Transcript distribution profile of SKR in different tissues of *Z. atratus* larvae. The data represent samples of *W* whole body, *B* brain, *VNC* ventral nerve cord, *FB* fat body, *G* gut, *H* haemolymph, *OE* oenocytes, *CC/CA* corpora cardiaca/corpora allata, *C* control (water)

Distribution of Zopat-SKR was analyzed directly in tissues using reverse transcription PCR (RT-PCR). Transcript level was diverse in different tissues, but we performed only spatial distribution analysis. The strongest bands intensity was observed in the nervous system (brain and ventral nerve cord), gut and oenocytes. Much lower level was observed in remaining tissues—fat body, haemolymph, CC/CA and in the whole body of larvae (Fig. 3). No foreign DNA or genomic DNA was found in the “no template control” and “no RT control”. We also used the reference gene—RpS18 as additional control in each analysis.

### The effect of sSK and nsSK on oenocytes lipid metabolism

**Fig. 4:**
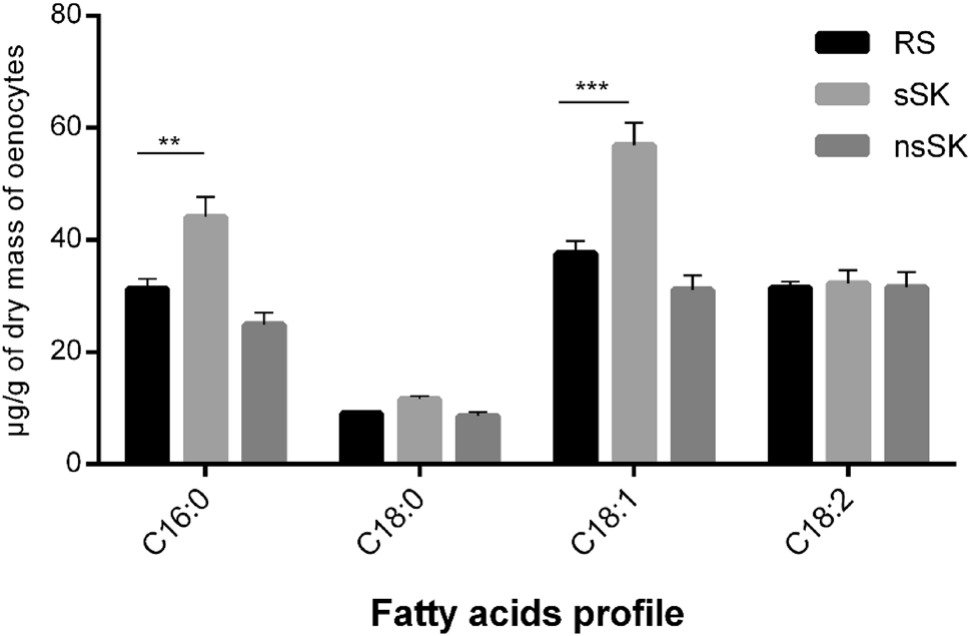
Contents of most abundant fatty acids (µg/g of oenocytes dry mass) found in the oenocytes of *Z. atratus* larvae treated with sulfated (sSK) or nonsulfated (nsSK) sulfakinins at doses of 20 pmol per larva. Control insects were injected with Ringer solution (RS). The oenocytes were collected 24 h after treatment. The data are shown as the mean ± SD, *n* = 20. Statistically significant differences (ANOVA) from the control values (RS) are indicated by asterisks as indicated: *p* ≤ 0.01 (**) or *p* ≤ 0.001 (***)

**Fig. 5:**
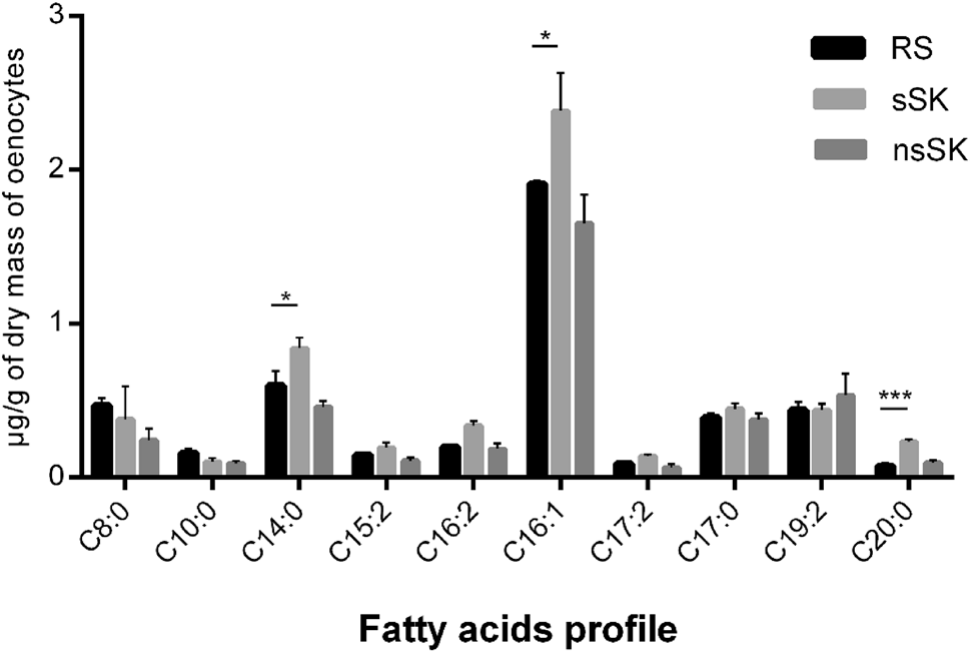
Contents of less abundant fatty acids (µg/g of oenocytes dry mass) found in the oenocytes of *Z. atratus* larvae treated with sulfated (sSK) or nonsulfated (nsSK) sulfakinins at doses of 20 pmol per larva. Control insects were injected with Ringer solution (RS). The oenocytes were collected 24 h after treatment. The data are shown as the mean ± SD, *n* = 20. Statistically significant differences (ANOVA) from the control values (RS) are indicated by asterisks as indicated: *p* ≤ 0.05 (*) or *p* ≤ 0.001 (***)

In the *Z. atratus* larval oenocytes, 14 fatty acids were present. They contained from 8 to 18 carbons in the chain (Figs. 4 and 5). The most abundant fatty acids (from 37.5 to 9 µg/g oenocytes dry mass) found in the control insects were oleic acid (C18:1), linoleic acid (C18:2), palmitic acid (C16:0) and stearic acid (C18:0) (Fig. 4). After injection of sulfated sulfakinin (sSK), the concentration of mentioned fatty acids significantly increased. The content of 18:1 and 16:0 increased more than 30% and these changes were statistically significant, *p* < 0.001—for oleic acid and *p* < 0.01—for palmitic acid. There were no changes in the content of linoleic acid in oenocytes of hormone-treated compared to control larvae. In addition, ten different saturated and unsaturated fatty acids presented in Fig. 5 were recorded in lower concentrations, with maximum of 2.4 µg/g of oenocytes dry mass. The content of unsaturated fatty acid 16:1 increased about 20% from 1.9 in control to 2.4 after sSK injection and in case of myristic fatty acid (C14:0) its content was even 25% higher than in the control group. The most pronounced changes were observed for arachidic acid (C20:0) which level increased twice after sSK treatment (*p* < 0.001). On the contrary, the application of nonsulfated sulfakinin (nsSK) caused a decrease of fatty acids content. The biggest drop was observed in palmitic acid, its amount changed from 31 down to 25 µg/g of oenocytes dry mass after nsSK injection in comparison to control. Also, oleic acid content was 17% lower in nsSK than in the control group. However, the observed changes were not statistically significant.

**Fig. 6:**
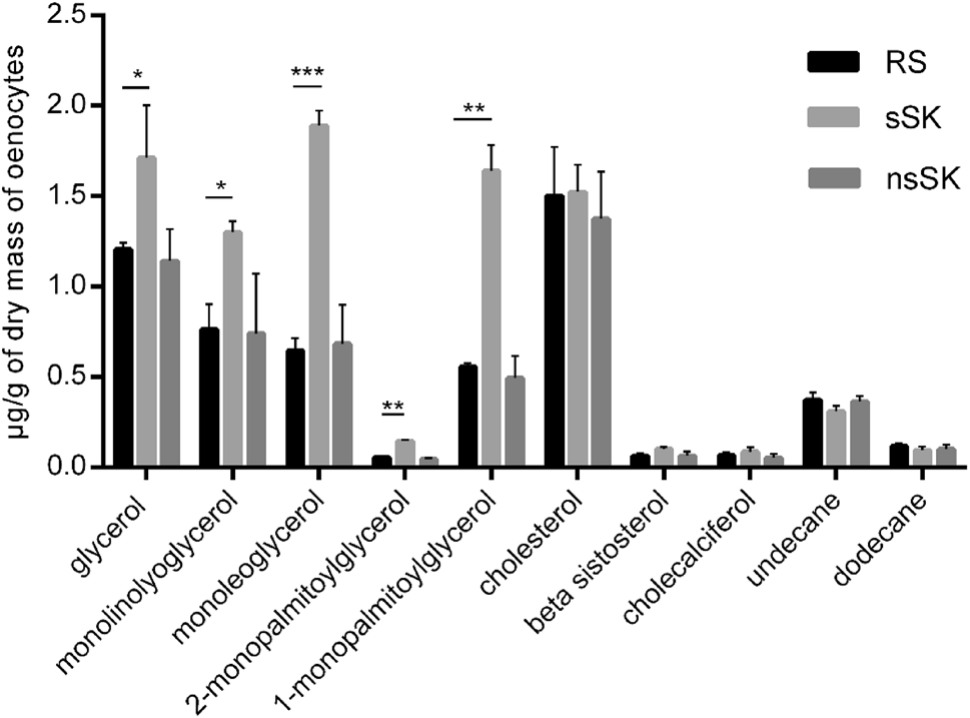
Contents of other compounds (µg/g of oenocytes dry mass) found in the oenocytes of *Z. atratus* larvae treated with sulfated (sSK) or nonsulfated (nsSK) sulfakinins at doses of 20 pmol per larva. Control insects were injected with Ringer solution (RS). The oenocytes were collected 24 h after treatment. The data are shown as the mean ± SD, *n* = 20. Statistically significant differences (ANOVA) from the control values (RS) are indicated by asterisks as indicated: *p* ≤ 0.05 (*), *p* ≤ 0.01 (**) or *p* ≤ 0.001 (***)

The analysis also included three different groups of other compounds: glycerol and its four derivatives, cholesterol and its two derivatives and two hydrocarbons (Fig. 6). From the first group, the monooleoglycerol was most abundant after sSK injection, and its level increased from 0.65 in control to 1.89 µg/g oenocytes dry mass and after hormone treatment. For 1-monopalmitoylglycerol increase of about 65% was observed. For both compounds, changes were statistically significant, *p* < 0.001 and *p* < 0.01, respectively. Similarly, statistical significance (*p* < 0.05) was observed after sSK application in monolinolyoglycerol and glycerol level, 42 and 27% more than in control group respectively. The least abundant from these groups was 2-monopalmitoylglycerol but the change in quantity of these compounds after sSK treatment was significant (*p* < 0.01) and its content increased from 0.055 up to 0.145 µg/g of oenocytes dry mass. The second group was mostly represented by cholesterol (ca. 1.5 µg/g) and less by β-sistosterol and cholecalciferol but the concentrations of those compounds after sulfakinins treatment were almost equal with the control group. To the third group, hydrocarbons, belonged undecane and dodecane. The level of these compounds did not change after sulfakinins injections.

**Fig. 7:**
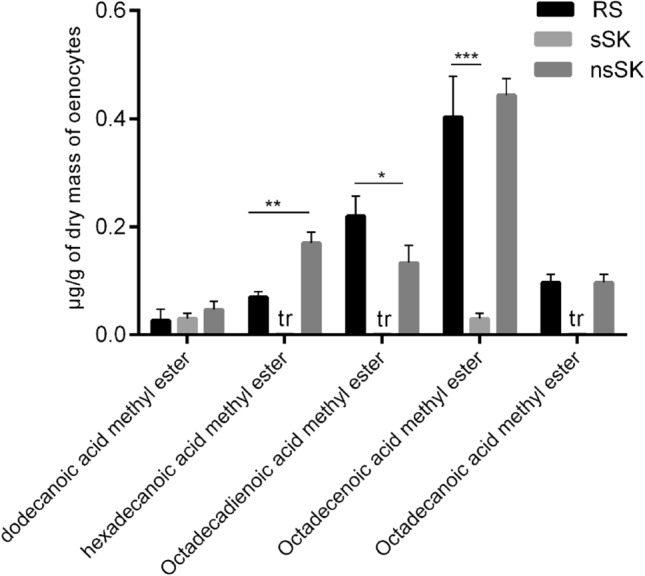
Total fatty acids methyl esters level (µg/g of oenocytes dry mass) in the larval oenocytes of control (RS) and sSK or nsSK injected insects. Sulfakinins were administrated at dose of 20 pmol sSK or nsSK per larvae. The oenocytes were collected 24 h after treatment. *Tr* trace amount. Statistically significant differences (ANOVA) from the control values (RS) are indicated by asterisks as indicated: *p* ≤ 0.05 (*), *p* ≤ 0.01 (**) or *p* ≤ 0.001 (***)

Oenocytes extracts obtained from the larvae of *Z. atratus* also contained five fatty acid methyl esters (FAME) from C:15 to C:19. The amount of FAME found in these samples are presented in Fig. 7. The most abundant FAME in the control group were esters of C:19 acids (octadecenoic acid ME, octadecadienoic acid ME and octadecanoic acid ME, respectively). After sSK injection the amount of FAME decreased, in some cases almost totally reduced to zero but only for octadecadienoic acid ME this change was statistically significant *(p* < 0.001). After nsSK application the content of C:17 (hexadecanoic acid ME) was even two times higher comparing to control and this change as well as for octadecadienoic acid ME was statistically significant, *p* < 0.01 and *p* < 0.05, respectively. For octadecenoic acid ME, the changes were not statistically significant. The same amount of FAME after nsSK injection and in the control group was observed in dodecanoic acid ME and octadecadienoic acid ME.

**Fig. 8:**
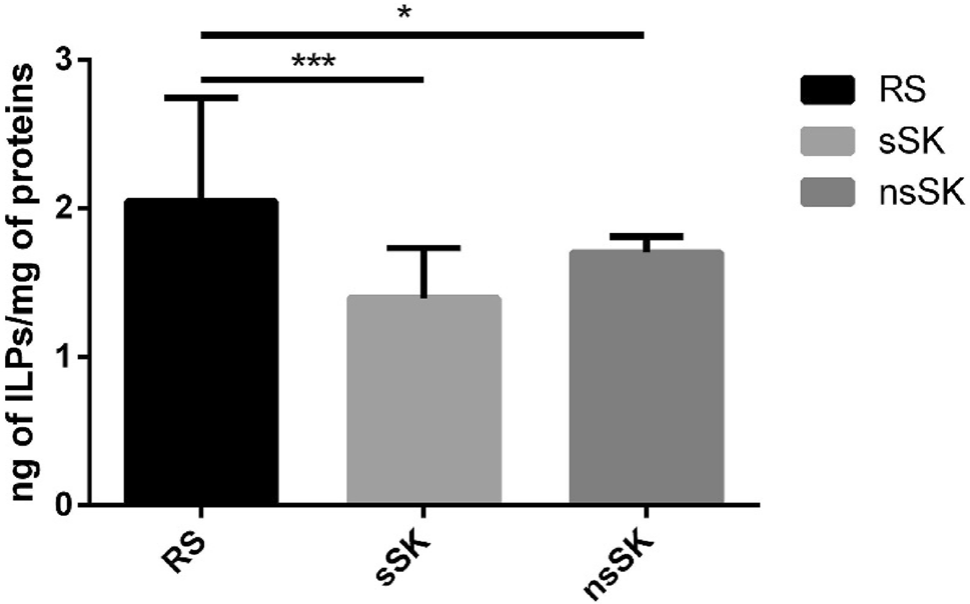
Content of ILPs (ng/mg of soluble protein) in the oenocytes of *Z. atratus* larvae after sulfated (sSK) and nonsulfated (nsSK) sulfakinins administration at doses of 20 pmol per larva. Control insects were injected with Ringer solution (RS). Oenocytes were collected 2 h after injection. The data are shown as the mean ± SD, *n* = 24. Differences (ANOVA) were considered as statistically significant if *p* ≤ 0.05 (*), and *p* ≤ 0.001 (***)

### The effect of sSK and nsSK on insulin-like peptides level in oenocytes

Using immunoenzymatic test, we determined the ILPs level (per mg of soluble proteins) in oenocytes. The average amount of ILPs in oenocytes, isolated from control insects, was 2.047 (± 0.698) ng/mg of proteins. Their level decreased significantly after injection of both sulfated and nonsulfated sulfakinin. The sSK lowered ILPs level about over 30% down to 1.395 (± 0.335); whereas, nsSK about 17% down to 1.702 (± 0.106) ng/mg of proteins (Fig. 8).

## Discussion

In the present study, we found evidence for important part of sulfakinins signaling in insect oenocytes. We characterized sulfakinin receptor (Zopat-SKR2) of *Z. atratus* in different tissues and organs of larvae particularly in the metabolic cells—oenocytes. We have examined the role of sSK and nsSK on the changes in fatty acids profiles in oenocytes of *Z. atratus* and determined the insulin-like peptides level in oenocytes after the hormones injections (Fig. 8).

In our study, the transcript profile of Zopat-SKR-2 indicated the strongest bands intensity in the nervous system (brain and ventral nerve cord), gut and for the first time in oenocytes while lower levels in fat body, haemolymph, and CC/CA. The presence of Zopat-SKR2 in oenocytes indicates a possible involvement of sulfakinins in the energy storing and releasing processes that take place in these tissues. Functions of sulfakinin are mediated by binding and signaling via G-protein coupled receptors with seven transmembrane helixes, the extracellular N-terminal tail and the cytoplasmic C-terminal segment for interactions with G proteins (Hauser et al. 2008). As was shown in the alignment (Fig. 1), the *Z. atratus* sulfakinin predicted receptor belongs to this family of receptors.

Our study reveals that insulin-like peptides level in isolated oenocytes decrease after sulfakinin application. The more significant decrease was observed after sSK and a bit less but still statistically significant was noted after nsSK injection. We used the ELISA test which is not without its drawbacks. It allows only to relate the total amount of different ILPs isoforms (between 1 and 38 in different insect species). ILPs are important regulators of metabolism, growth, reproduction and lifespan (Nässel and Vanden Broeck 2016). The observed changes in the ILPs levels in the oenocytes of *Z. atratus* possibly change the biochemical compound content in the cooperating organs and tissues in the insect body—oenocytes, fat body, midgut, and haemolymph. To confirm this balance between different parts of the body we can compare our results with the results of Słocińska et al. (2020). They injected *T. molitor* larvae with 20 pmol of sSK or nsSK and measured the concentrations of ILPs in the haemolymph 2 h after hormone treatment. Both sSK and nsSK induced an increase in ILPs concentration. In contrary, in case of *Z. atratus* larvae oenocytes, we observed a decrease of ILPs level after the same dose of sulfakinin. Nevertheless, further work is required to understand feedback between ILPs and circulating sulfakinins that regulate feeding and metabolism.

The interplay between SKs and ILPs have been studied also in *D. melanogaster* where it was shown that SK and ILPs were expressed in fly insulin producing cells (IPCs) indicating that they are likely to cooperate in regulation of feeding and metabolism (Chowański et al. 2021; Söderberg et al. 2012). It was shown that knockdown of either neuropeptide affects the transcript levels of the other, suggesting a possible feedback regulation between the SKs and ILPs (Söderberg et al. 2012). Flies with impaired ILPs showed a decrease in *SKs* expression level in normal feeding adults whereas knockout of *SK* increased the ILPs expression which might be in agreement with our studies (Söderberg et al. 2012). However, the mechanisms underlying interplay between central and peripheral signaling might not be common.

Our results confirmed the presence of the main fatty acids (FA) in oenocytes involved in the synthesis of triacylglycerols and diacylglycerols (DAG). The dominant FAs found in oenocytes of *Z. atratus* larvae were oleic (18:1); linoleic (18:2); palmitic (16:0); stearic (18:0) acids (Fig. 4). TAGs are a major lipid class in the fat body and act as the main source of energy for insects (Canavoso et al. 2001). Słocińska et al. (2019) observed an increase in larval fatty acids content in the fat body and haemolymph after nonsulfated sulfakinin injections. Our results show opposite results in oenocytes after nsSK but the strongest effects were noticed after sSK injections for oleic and palmitic acid. We observed also changes in unsaturated fatty acids level, the level of the 16:1 and 14:0 after sSK application was about 20 and 25% higher in comparison to the control group, respectively. Arachidic acid (20:0) belonging to saturated FA revealed the double increased level after sSK treatment. The increase of FA level in larvae oenocytes of *Z. atratus* indicates activation of lipid stores by sulfated sulfakinins. In case of nsSK injections, we observed decrease of FA content in isolated oenocytes of *Z. atratus*. These results suggest for strong interactions between fat body, haemolymph and oenocytes.

We also found glycerol and cholesterol and their derivatives and two hydrocarbons in the oenocyte extracts (Fig. 6.). The amount of glycerol, monolinolyoglycerol, monooleoglycerol, 1-monopalmitoylglycerol, monopalmitoylglycerol increased significantly after sSK treatment and remained stable after nsSK. It indicates the importance of sulfation moiety in the peptide structure for regulation of lipid compounds level in oenocytes. The concentrations increase after sSK possibly because of hydrolysis of TAG. Słocińska et al. (2019) reported almost doubled the amount of glycerol in the haemolymph after nsSK applications which is contrary to our data. It may suggest influence of SKs on interactions between oenocytes and haemolymph. The sterols detected in oenocytes of *Z. atratus* larvae were cholesterol, beta sistosterol and cholecalciferol, the former being predominant. Cholesterol is required for insect development and growth, but insects are unable to synthesize it de novo.

## Conclusions

Our study indicated for the first time the presence of sulfakinin receptor in *Z. atratus* larvae and for the first time Zopat-SKR transcript in insects oenocytes. We demonstrated the influence of sulfakinins on the fatty acid profile and concentrations of lipid metabolites in larvae of *Z. atratus*. The level of particular fatty acid and lipid compounds is regulated by sulfakinins what indicate for their important role in the regulation of intermediary metabolism in the larval body. We have demonstrated also that sulfakinins regulate ILPs level in oenocytes. We still need more research about the connections between oenocytes, fat body and haemolymph to understand their role in insect metabolism.

## Supplementary Information

Below is the link to the electronic supplementary material.

Supplementary file1 (DOCX 66 KB)

## Declarations

### Availability of data and material

Data are available on request.

### Code availability

AMU license.
