# Supplementary material for: Sulfakinins influence lipid composition and insulin-like peptides level in oenocytes of Zophobas atratus beetles
Source: J Comp Physiol B. 2021 Aug 20;192(1):15–25. doi: 10.1007/s00360-021-01398-2 (PMC8816747; doi:10.1007/s00360-021-01398-2)

**Supplementary Fig. 1.**

Protein structure model of Z. *atratus* SKR. The schematic representations indicate the orientation of the receptor protein and seven transmembrane domains. Predicted glycosylation sites are highlighted in orange, in purple putative phosphorylation sites and in green predicted palmitoylation sites.


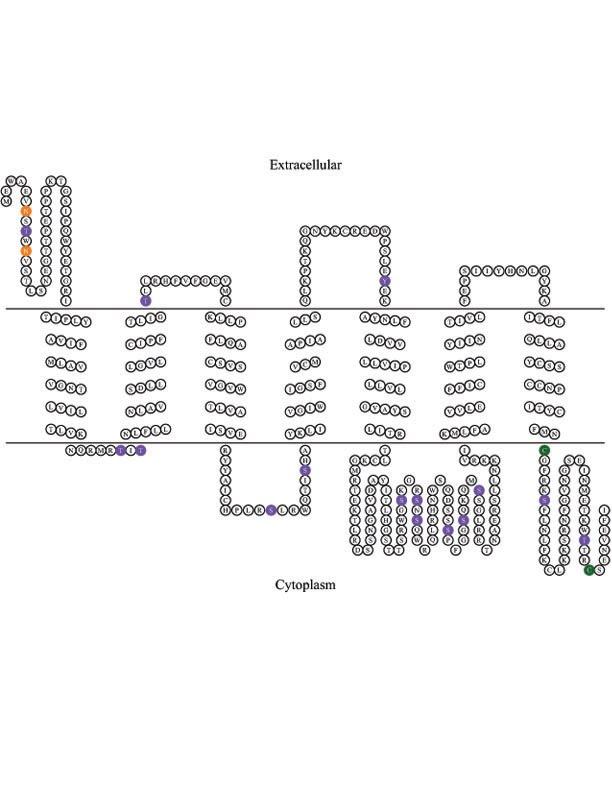

Supplement: Supplementary file 1 — Supplementary file1 (DOCX 66 KB) [file 360_2021_1398_MOESM1_ESM.docx]
